# Predicting return to work after long-term sickness absence with subjective health complaints: a prospective cohort study

**DOI:** 10.1186/s12889-020-09203-5

**Published:** 2020-07-11

**Authors:** Kristel H. N. Weerdesteijn, Frederieke Schaafsma, Karin Bonefaas-Groenewoud, Martijn Heymans, Allard Van der Beek, Johannes Anema

**Affiliations:** 1 Department of Public and Occupational Health, Amsterdam Public Health Research Institute, Amsterdam UMC, Vrije Universiteit Amsterdam, Van der Boechorststraat 7, 1081 BT Amsterdam, The Netherlands; 2 Research Center for Insurance Medicine (KCVG), AMC-UMCG-UWV-VUmc, PO Box 7057, 1007 MB Amsterdam, The Netherlands; 3 Department of Social Medical Affairs (SMZ), The Dutch Social Security Institute: the Institute for Employee Benefits Scheme (UWV), La Guardiaweg 94-114, 1043 DL Amsterdam, the Netherlands; 4 Department of Epidemiology and Biostatistics, Amsterdam Public Health Research Institute, Amsterdam UMC, Vrije Universiteit Amsterdam, Van der Boechorststraat 7, 1081 BT Amsterdam, The Netherlands

**Keywords:** Medically unexplained symptoms, Predictor, Prognostic factor, Rehabilitation, Sick-listed

## Abstract

**Background:**

Long-term sickness absence results in increased risks of permanent disability and a compromised quality of life. Return to work is an important factor in reducing these risks. Little is known about return to work factors for long-term sick-listed workers with subjective health complaints. The aim of this study was to evaluate prognostic factors for partial or full return to a paid job for at least 28 days for long-term sick-listed workers with subjective health complaints, and to compare these factors with those of workers with other disorders.

**Methods:**

Data from a prospective cohort study of 213 participants with subjective health complaints and 1.037 reference participants were used. The participants answered a questionnaire after 84 weeks of sickness absence. Return to work was measured after one and two years. Univariable logistic regression analyses were performed (*P* ≤ 0.157) for variables per domain with return to work (i.e. demographic, socio-economic and work-related, health-related, and self-perceived ability). Subsequently, multivariable logistic regression analyses with backward selection (*P* ≤ 0.157) were performed. Remaining factors were combined in a multivariable and final model (*P* ≤ 0.05).

**Results:**

Both for workers with subjective health complaints and for the reference group, non-health-related factors remained statistically significant in the final model. This included receiving a partial or complete work disability benefit (partial: OR 0.62, 95% CI 0.26–1.47 and OR 0.69, 95% CI 0.43–1.12; complete: OR 0.24, 95% CI 0.10–0.58 and OR 0.12, 95% CI 0.07–0.20) and having a positive self-perceived possibility for return to work (OR 1.06, 95% CI 1.01–1.11 and OR 1.08, 95% CI 1.05–1.11).

**Conclusions:**

Non-health-related factors seem to be more important than health-related factors in predicting return to work after long-term sickness absence. Receiving a work disability benefit and having negative expectations for return to work seem to complicate return to work most for workers with subjective health complaints. With respect to return to work predictors, workers with subjective health complaints do not differ from the reference group.

## Introduction

Long-term sickness absence is of great concern in the developed industry because of high productivity loss, and high compensation and treatment costs [1]. While most workers return to work (RTW) within the first months of sickness absence, one-third of sick-listed workers remain absent for a much longer period of time [1, 2]. The leading causes for long-term sickness absences are chronic disorders, based on mental, musculoskeletal and cardio-vascular health complaints [3]. Most of these health complaints can be explained by well-defined diseases; however, there are also persistent subjective health complaints (SHC) that cannot be fully explained by such well-defined diseases [4]. SHC refer to symptoms (e.g. fatigue, pain, dizziness) and syndromes (e.g. fibromyalgia, irritable bowel syndrome), for which no clear organic cause is currently found after appropriate medical examination. SHC are identical to other common terms, such as medically unexplained physical symptoms (MUPS) or persistent physical symptoms (PPS), which also refer to complaints with an unknown underlying pathology.

Research has suggested that long-term sick-listed workers with SHC have an increased risk of permanent disability, a weakened financial position, social isolation and a compromised quality of life [5, 6]. RTW is an important factor in reducing these economic, societal and personal consequences. In most European countries, physicians have to support sick-listed workers in their RTW process [7]. Physicians, however, have reported difficulties in supporting the RTW process of sick-listed workers with SHC in particular, due to the lack of objective medical findings and limited knowledge on relevant factors in long-term sickness absence and RTW for workers with SHC [8].

Most studies on long-term sickness absence and RTW have been performed for workers with well-defined diseases, specific physical symptoms or across several health conditions [9–12]. These studies have revealed that health-related factors, such as the severity of the disease and the symptoms, seem to become less relevant for RTW in long-term sickness absence than for RTW in short-term sickness absence [9–12]. External and psychosocial factors, such as self-perceived health and disability, job demands and strain, claim-related aspects, age, self-efficacy and own expectations for RTW seem to become more important for RTW in the later phases of sickness absence [9–12]. This suggests that the RTW process after long-term sickness absence benefits from a more phase-specific and multifactorial approach across several health conditions.

To date, little attention has been devoted to determine RTW factors for long-term sick-listed workers with SHC, and the evidence that is available is conflicting and of low quality [13]. More knowledge of factors on RTW after long-term sickness absence for workers with SHC is highly relevant for physicians to better identify sick-listed workers with SHC and to better support these workers in their RTW process. Medico-legal criteria on which disability systems are often based together with the lack of objective medical findings can make it difficult to use SHC to claim work disability benefits [14, 15]. The system in the Netherlands, in which a well-defined medical disease is not a prerequisite for a work disability benefit, provides an unique opportunity to analyse relevant prognostic factors for RTW for long-term sick-listed workers with SHC [16].

This study was designed to evaluate the prognostic factors for RTW for workers with SHC after long-term sickness absence (> 84 weeks) and to compare these factors with the prognostic factors for RTW for long-term sick-listed workers with other disorders as a reference group. We believe that understanding the most relevant factors for RTW for long-term sick-listed workers with SHC can help reduce sickness absence among these workers and optimise their rehabilitation and RTW process. Our results will give physicians more insight into whether they should give comparable advice and suggest comparable interventions for RTW for long-term sick-listed workers with SHC and for those with other disorders.

## Materials and methods

### Study population and design

This study used data from the Forward study, a Dutch longitudinal cohort of 2593 out of 44,379 long-term sick-listed workers aged 18–65 years, who had been registered as sick-listed for at least 84 weeks between June 2014 and May 2015 in the electronic database of The Dutch Social Security Institute (UWV). The participants of Forward did not meet the exclusion criteria of Forward (unable to fill in questionnaires; no longer sick-listed; hospitalised; involved in judicial procedures; pregnant in the three months before study entry; suffering from cancer, a psychotic disease or dementia in the twelve months before study entry; and a PHQ-15 score of ≤5) [17]. The Forward study followed the included participants for 24 months after baseline, and measurements with questionnaires were taken at baseline (T0), after one year (T1) and after two years (T2). Further information about the study population of Forward has been described comprehensively elsewhere [18].

**Fig. 1:**
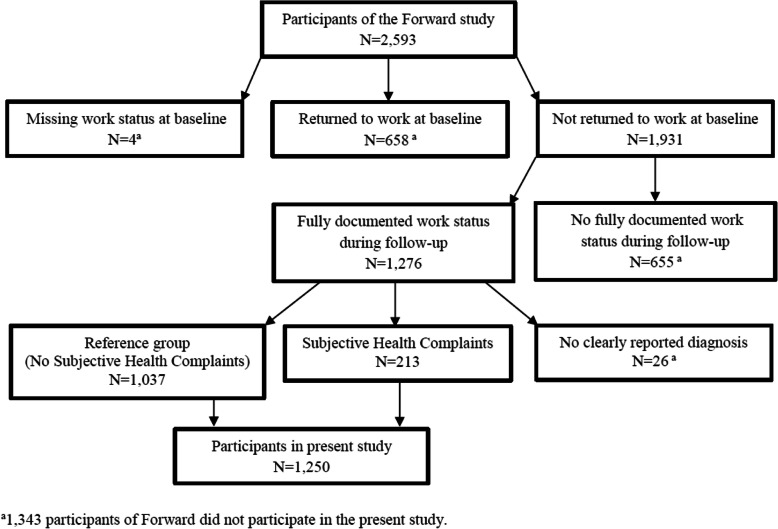
Flow chart of the present study population

Figure 1 shows the flowchart of the study sample for the present study. The present study selected 1250 participants out of the 2593 Forward participants. Participants were included if they were not returned to work at baseline, their work status had been fully documented in the questionnaires during follow-up and if they were clearly diagnosed with SHC (subjective health complaints) or another disorder. Information about diagnoses was derived from medical work disability assessment data of UWV. In the Netherlands, workers who are sick listed for at least 84 weeks can apply for a medical work disability assessment at UWV. These assessment result in a diagnosis by an insurance physician (IP) based on the International Classification of Diseases (ICD classification) [19]. IPs can report 10 functional somatic syndromes: Chronic Fatigue syndrome, Fibromyalgia, Irritable Bowel Syndrome, Pelvic Girdle Pain, Repetitive Strain Injury, Somatic (Pain) Syndrome, Somatization disorder, Tension Headache, Tietze Syndrome and Whiplash [20]. IPs can also report one of the 25 functional somatic symptoms that match with the 23 (partially) unexplained physical complaints of the Robbins list [21]. For this study, participants were defined as suffering from SHC if the IP reported a functional somatic syndrome or symptom. All other participants with a clear diagnosis were defined as the reference group.

### Measures

#### Dependent variable

The primary outcome measure was RTW (return to work) during follow-up, with RTW defined as a partial or full return to a paid job for a duration of at least 28 days. This outcome measure was based on self-reported answers to the follow-up questionnaires at T1 and T2. The answer options in the questionnaire were:

- partial or full return to my usual job or another paid job for ≥28 days;
- partial or full return to my usual job or another paid job for < 28 days;
- no return to a paid job;
- no return to a job at all.

Participants who had partially or fully returned to their usual or another paid job for < 28 days or who had not returned to a paid job or any job at all were combined into one category.

#### Independent variables

The selection of independent variables was based on literature regarding predictors for RTW and work ability outcomes in general [9–12]. The selected variables were divided into domains based on the biopsychosocial model. This is a universal, well-known conceptual framework that focuses on health conditions and internal and external contextual factors. It was chosen for this study as it is useful for assessing all aspects of disability and functioning [15, 22]. The independent variables were classified into the following four domains:

1. Demographic
2. Socio-economic and work-related
3. Health-related
4. Self-perceived ability

All variables were collected at baseline via self-reported questionnaires, except for the variable work disability benefits in the socio-economic and work-related domain, which was derived from UWV data after the medical work disability assessment.

##### Demographic domain

The demographic domain included answers to questions about age (years), gender (male/female), marital or partner status (yes/no), breadwinner of the family (yes/no), land of birth (The Netherlands or another country), and educational level (primary school/secondary school/high school/bachelor and master).

##### Socio-economic and work-related domain

The socio-economic and work-related domain contained answers to questions about the participants job and work status: collar job (blue/white/pink), being employed (yes/no), usual working time (hours), regular work schedule (yes/no), managerial position (yes/no), job demands (physical/psychological/combination of both), and previous absenteeism for the same reason (yes/no). This domain also contained information about work disability benefits (no/partial/complete) and about stressors and support, based on answers to the following validated questionnaire:

- The Work and Well-Being Inventory (WBI) questionnaire. The stressors and support variables were based on two subscales of the WBI. The stressors subscale contains 16 questions, with a scoring range between 16 and 64 (higher scores indicate more stressors); the support subscale contains 21 questions, with a scoring range between 21 and 84 (higher scores indicate better or more support) [23].

##### Health-related domain

The health-related domain included answers to questions about the use of specialist or psychiatric care in the last two years (yes/no) and the use of medication (yes/no). It also included information on the presence of a depressive or anxiety disorder, the severity of complaints, the physical and mental health, the presence of hypochondria, and symptom scale and coping strategies, based on answers to the following validated questionnaires:

- The Hospital Anxiety and Depression Scale (HADS). The presence of a depressive or anxiety disorder was assessed by using two subscales of the HADS. Each subscale contains seven questions about the presence of a depressive or anxiety disorder, with a scoring range of 0–21 for each separately. Scores of ≤7 mean no disorder (no), scores between 8 and 10 mean a possible disorder (maybe), and scores of ≥11 mean a definite disorder (yes) [24].
- The Patient Health Questionnaire (PHQ-15). The severity of complaints was based on the PHQ-15. This questionnaire contains 15 questions about the severity of complaints, with a scoring range of 5–30. Scores between 5 and 10 represent mild somatic complaints, scores between 10 and 15 represent moderate somatic complaints, and scores of ≥15 represent severe somatic complaints [17].
- The Short Form Health Survey 36 (SF-36). The physical and mental health (PCS and MCS) and the health change (SF-2) were measured by using the SF-36. PCS and MCS were measured by using a validated formula on total scores of the SF-36. The scoring range lies between 0 and 100 for each score separately, with higher scores indicating better levels of mental and physical health and functioning. The SF-2 was compiled from the following question on the SF-36: “How is your health in general compared with a year ago?” We categorised the five answering options of SF-2 into three categories: one category with the answers ‘much better’ and ‘somewhat better’ (better), one category with the answer ‘no difference’ (same), and one category with the answers ‘somewhat worse’ and ‘much worse’ (worse) [25, 26].
- The Whitely Index questionnaire (WI). The presence of hypochondria was measured with the WI. This questionnaire contains 14 questions, with a scoring range between 0 and 14. Scores between 0 and 8 mean ‘no hypochondria’ (no) and scores of ≥8 mean ‘definitely hypochondria’ (yes) [27].
- The WBI questionnaire. The symptom scale and coping strategies were based on two subscales of the WBI. The subscale about the symptom scale contains 20 questions, with a scoring range between 20 and 80 (a higher score means a higher risk for symptoms); the subscale about coping strategies contains 21 questions, with a scoring range between 17 and 68 (a higher score means less coping) [23].

##### Self-perceived ability domain

The self-perceived ability domain contained one answer to a question about RTW expectations (yes or maybe/no). It also contained answers to the following validated questionnaires about disability, work ability in general and in the context of work load, and possibilities for RTW.

- The WBI questionnaire. Self-perceived disability was based on the disability subscale of the WBI. The subscale contains seven questions, with a scoring range between 7 and 28. Higher scores on this subscale mean more self-perceived disability [23].
- The Work Ability Index (WAI). Perception of work ability in general and in the context of work load were both derived from the WAI. The category about work ability in general, also called The Work Ability Score (WAS), contains one question, with a scoring range between 0 and 10. The category about work ability in the context of work load contains two questions, with a scoring range between 2 and 10. For both categories higher scores indicate higher self-perceived work ability [28].
- The Obstacles to Return to Work Questionnaire (ORQ). The self-perceived possibilities for RTW were derived from the subscale “Perceived Prognosis of Work Return” of the ORQ. This subscale contains six questions, with a scoring range between 0 and 36. Higher scores mean higher self-perceived possibilities for RTW [29].

### Statistics

For the analyses, participants were divided into two subgroups: workers with SHC and those with other disorders as the reference group. All further analyses were performed for both groups separately. Firstly, descriptive analyses were used to describe both groups at baseline. Secondly, to obtain information about possible predictors for RTW, univariable logistic regression analyses were performed for all independent variables per domain separately (i.e. demographic, socio-economic and work-related, health-related, and self-perceived ability), with the dependent variable partial or full RTW to a paid job for ≥28 days.

A cut-off *p*-value ≤0.157 [30] was used for the univariable analyses. Multicollinearity between the variables was checked. Multicollinearity was assumed if the analyses showed variance inflation factor (VIF) scores of ≥10 [31]. Variables that had a *p*-value ≤0.157 in the univariable analyses and a VIF score of < 10 in the correlation analyses were included in a combined multivariable logistic regression analysis with backward selection per domain separately. In the next step, all variables that had a *p*-value ≤0.157 in the combined models per domain were included in one multivariable model. Odds ratios (OR) and 95% confidence intervals (95% CI) were calculated to show associations with RTW in this multivariable model. Subsequently, variables with a p-value ≤0.05 were combined in a final model. The Hosmer and Lemeshow test was performed and the Nagelkerke’s R2 was assessed to measure the overall fit and the overall predictive ability of the final model [31].

The analyses were based on complete case analyses. In complete case analyses, missing data may give bias due to selective loss to follow-up. To explore the robustness of the complete case analyses, missing data sensitivity analyses were also performed by using a multiple imputation approach [32]. The analyses were identical for both approaches. SPSS version 24.0 and R-studio were used for all statistical analyses.

## Results

Baseline characteristics of the participants with SHC (subjective health complaints) (*n* = 213) and of the reference group (*n* = 1037) are shown separately in Table 1. On average, the participants with SHC were more often women, less often the breadwinner of the family, usually worked fewer hours and received less complete work disability benefits than the reference group (Table 1).

**Table 1:** Baseline characteristics of the study population

Domains		SHC^a^ (N = 213)		Other disorders (N = 1037)	
	Categories/Ranges	Mean/N^b^	SD^c^/%	Mean/N	SD/%
***Demographic***					
Age in years	18–34	16	8%	54	5%
	35–44	36	17%	126	12%
	45–54	75	35%	350	34%
	55–65	86	40%	507	49%
Gender	Male	76	36%	561	54%
Marital status	Married or partner	152	71%	777	75%
Breadwinner of the family	Yes	127	60%	676	65%
Land of birth	The Netherlands	193	91%	947	91%
Educational level	None/Primary school	24	11%	87	8%
	Secondary school	78	37%	419	41%
	High school	68	32%	335	32%
	Bachelor/Master	43	20%	194	19%
***Socio-economic and work-related***					
Collar job	Blue	60	29%	337	34%
	White	87	42%	336	34%
	Pink	61	29%	316	32%
Employer	Yes	77	36%	366	37%
Usual working time in hours	4–60	31.64	10.34	32.69	10.91
Work schedule	Regular	144	68%	664	64%
Managerial position	Yes	42	20%	227	22%
Job demands	Psychological	48	23%	244	24%
	Physical	77	36%	330	32%
	Psychological and physical	88	41%	456	44%
Stressors	16–64	38.56	9.44	38.27	9.25
Support	21–84	58.20	13.02	58.90	12.73
Previous absenteeism same reason	Yes	109	51%	479	47%
Work disability benefit	No	51	26%	144	14%
	Partial	50	25%	257	26%
	Complete	99	49%	600	60%
***Health-related***					
Use of specialist care last 2 years	Yes	191	90%	879	85%
Use of psychiatric care last 2 years	Yes	120	56%	486	47%
Use of medication	Yes	196	92%	922	89%
Depressive disorder	No	68	32%	383	37%
	Maybe	50	24%	233	23%
	Yes	94	44%	419	40%
Anxiety disorder	No	82	39%	412	40%
	Maybe	44	21%	244	23%
	Yes	86	40%	381	37%
Severity of complaints	Mild	39	18%	344	33%
	Moderate	56	26%	362	35%
	Severe	118	56%	331	32%
Physical Health	0–100	29.73	8.89	31.57	9.79
Mental Health	0–100	33.32	12.58	34.68	13.81
Health change comparing last year	Worse	124	58%	556	54%
	Same	51	24%	306	29%
	Better	37	18%	174	17%
Hypochondria	Yes	155	73%	706	68%
Symptom scale	20–80	46.60	11.16	44.94	12.05
Coping strategies	17–68	43.40	9.77	42.70	9.52
***Self-perceived ability***					
Return to work expectation	Yes or maybe	148	70%	688	66%
Disability	7–28	25.31	3.50	24.84	3.64
Work ability in general	0–10	1.99	1.78	2.20	1.95
Work ability in context of work load	0–10	4.13	1.50	4.28	1.62
Possibilities for returning to work	0–36	9.83	7.81	9.53	8.15

^a^SHC = Subjective Health Complaints

^b^N = Number

^c^SD = Standard Deviation

### RTW (return to work) predictors for participants with SHC

Of the 213 participants with SHC, 47 participants (22%) returned to work. For RTW after two years of sickness absence we found significant univariable associations (*P* ≤ 0.157) in the domains demographic, socio-economic and work-related, health-related and self-perceived ability (Table 2). We found no multicollinearity for any of the variables in the domains (data not shown). We used backward selection and further select one or two variables with a *P*-value ≤0.157 in all four domains, which we combined in a multivariable analysis. One variable in the socio-economic and work-related domain and one variable in the self-perceived ability domain remained statistically significant (*P* ≤ 0.05) (Table 3), which we combined in a final multivariable model. In the final model, we found that the chance of RTW after two years of sickness absence decreased if participants obtained a partial (OR 0.62, 95% CI 0.26–1.47) or a complete (OR 0.24, 95% CI 0.10–0.58) work disability benefit after these two years. In addition, we found that a higher self-perceived possibility for RTW increased the chance for RTW after two years of sickness absence (OR 1.06, 95% CI 1.01–1.11). The Hosmer and Lemeshow test was not statistically significant (*P*-value 0.19), indicating that there was a good fit of the final model, and the Nagelkerke’s R2 was 0.22.

**Table 2:** Univariable logistic regression analyses of potential predictors for participants with SHC^a^ and other disorders separately

Domains		SHC^a^ (N = 213)		Other disorders (N = 1037)	
	Categories/Ranges	OR^b^	95% CI^c^	OR	95% CI
***Demographic***					
Age in years	18–34	Reference		**Reference**^d^	
	35–44	0.48	0.13–1.72	**0.71**	**0.36–1.37**
	45–54	0.32	0.10–1.04	**0.52**	**0.29–0.95**
	55–65	0.54	0.18–1.66	**0.23**	**0.12–0.42**
Gender	Male	Reference		Reference	
	Female	0.87	0.44–1.69	0.87	0.64–1.18
Married or partner	No	Reference		Reference	
	Yes	0.82	0.41–1.65	0.99	0.70–1.41
Breadwinner of the family	No	Reference		Reference	
	Yes	1.41	0.72–2.78	1.10	0.80–1.52
Land of birth	The Netherlands	**Reference**		**Reference**	
	Other country	**2.63**	**1.01–6.88**	**1.48**	**0.90–2.42**
Educational level	None/Primary school	**Reference**		**Reference**	
	Secondary school	**1.27**	**0.33–4.94**	**1.86**	**0.89–3.87**
	High school	**2.71**	**0.73–10.17**	**2.76**	**1.33–5.76**
	Bachelor/Master	**3.03**	**0.77–11.98**	**2.77**	**1.29–5.95**
***Socio-economic and work-related***					
Collar job	Blue	Reference		Reference	
	White	1.01	0.46–2.24	0.99	0.68–1.43
	Pink	1.08	0.46–2.54	0.99	0.68–1.44
Employer	No	Reference		Reference	
	Yes	1.12	0.57–2.20	1.17	0.86–1.60
Usual working time in hours	4–60	1.00	0.97–1.03	**1.01**	**1.00–1.03**
Work schedule	Irregular	Reference		Reference	
	Regular	0.81	0.41–1.59	0.94	0.69–1.28
Managerial position	No	Reference		**Reference**	
	Yes	1.13	0.51–2.51	**1.72**	**1.22–2.41**
Job demands	Psychological	Reference		Reference	
	Physical	0.67	0.28–1.60	0.81	0.54–1.21
	Psychological and physical	0.94	0.42–2.13	0.88	0.60–1.28
Stressors	16–64	0.99	0.95–1.02	1.01	0.99–1.03
Support	21–84	1.00	0.98–1.03	**0.99**	**0.98–1.00**
Previous absenteeism same reason	No	Reference		Reference	
	Yes	1.10	0.57–2.10	0.92	0.68–1.25
Work disability benefit	No	**Reference**		**Reference**	
	Partial	**0.59**	**0.25–1.38**	**0.62**	**0.41–0.94**
	Complete	**0.19**	**0.08–0.45**	**0.08**	**0.05–0.12**
***Health-related***					
Use of specialist care last 2 years	No	Reference		**Reference**	
	Yes	0.96	0.33–2.75	**0.54**	**0.37–0.79**
Use of psychiatric care last 2 years	No	Reference		Reference	
	Yes	1.06	0.55–2.04	1.13	0.84–1.53
Use of medication	No	Reference		**Reference**	
	Yes	2.24	0.49–10.14	**0.44**	**0.29–0.67**
Depressive disorder	No	Reference		Reference	
	Maybe	0.42	0.16–1.10	1.45	0.98–2.15
	Yes	0.74	0.36–1.52	1.12	0.79–1.60
Anxiety disorder	No	Reference		Reference	
	Maybe	1.19	0.50–2.80	1.05	0.70–1.57
	Yes	0.94	0.45–1.97	1.23	0.87–1.74
Severity of complaints	Mild	Reference		**Reference**	
	Moderate	1.29	0.48–3.46	**0.83**	**0.58–1.19**
	Severe	1.04	0.43–2.55	**0.66**	**0.45–0.97**
Physical Health	0–100	**1.03**	**0.99–1.06**	**1.05**	**1.03–1.06**
Mental Health	0–100	1.01	0.99–1.04	1.00	0.98–1.01
Health Change comparing last year	Worse	**Reference**		**Reference**	
	Same	**0.78**	**0.32–1.86**	**1.80**	**1.26–5.57**
	Better	**2.84**	**1.29–6.28**	**3.13**	**2.12–4.64**
Hypochondria	No	**Reference**		Reference	
	Yes	**0.57**	**0.28–1.13**	0.92	0.66–1.26
Symptom scale	20–80	0.98	0.95–1.01	1.00	0.99–1.02
Coping strategies	17–68	1.00	0.97–1.04	1.00	0.98–1.01
***Self-perceived ability***					
Return to work expectation	No	**Reference**		**Reference**	
	Yes or maybe	**2.42**	**1.06–5.53**	**3.47**	**2.32–5.19**
Disability	7–28	**0.91**	**0.84–0.99**	**0.86**	**0.83–0.89**
Work ability in general	0–10	**1.30**	**1.09–1.56**	**1.35**	**1.25–1.46**
Work ability in the context of work load	0–10	**1.26**	**1.01–1.57**	**1.34**	**1.22–1.47**
Possibilities for returning to work	0–36	**1.08**	**1.04–1.12**	**1.13**	**1.11–1.15**

^a^SHC = Subjective Health Complaints

^b^OR = Odds ratio

^c^95% CI = 95% confidence intervals

^d^Numbers in bold had a p-value of ≤0.157

**Table 3:** Multivariable logistic regression analysis of all predictors for participants with SHC^a^ and other disorders separately

Domains		SHC^a^ (N = 213)		Other disorders (N = 1037)	
	Categories/Ranges	OR^b^	95% CI^c^	OR	95% CI
***Demographic***					
Age in years	18–34			**Reference**^d^	
	35–44			**0.91**	**0.39–2.16**
	45–54			**0.72**	**0.32–1.61**
	55–65			**0.39**	**0.17–0.88**
Land of birth	The Netherlands	Reference		Reference	
	Another country	2.61	0.88–7.77	1.14	0.61–2.15
Educational level	None/Primary school	Reference		Reference	
	Secondary school	1.13	0.26–4.95	1.25	0.52–2.99
	High school	1.95	0.45–8.40	1.71	0.71–4.07
	Bachelor/Master	2.22	0.49–10.12	1.52	0.60–3.82
***Socio-economic and work-related***					
Usual working time in hours	4–60			1.01	0.99–1.03
Manegerial position	No			**Reference**	
	Yes			**1.56**	**1.00–2.45**
Work disability benefit	No	**Reference**		**Reference**	
	Partial	**0.71**	**0.29–1.78**	**0.66**	**0.40–1.08**
	Complete	**0.26**	**0.10–0.66**	**0.12**	**0.07–0.20**
***Health-related***					
Use of medication	No			Reference	
	Yes			0.88	0.51–1.50
Physical Health	0–100			1.00	0.98–1.03
Health Change comparing last year	Worse	Reference		Reference	
	Same	0.51	0.18–1.45	0.98	0.63–1.54
	Better	1.31	0.49–3.51	0.84	0.48–1.48
***Self-perceived ability***					
Work ability in general	0–10			**1.11**	**1.00–1.24**
Possibilities for returning to work	0–36	**1.05**	**1.00–1.11**	**1.08**	**1.05–1.11**

^a^SHC = Subjective Health Complaints

^b^OR = Odds ratio

^c^95% CI = 95% confidence intervals

^d^Numbers in bold had a p-value of ≤0.05 and were combined the final model

### RTW predictors for the reference group (participants with other disorders than SHC)

In the reference group (*n* = 1037), 211 participants (20%) returned to work. We found significant univariable associations (*P* ≤ 0.157) in all four domains for RTW (Table 2), and no multicollinearity in any of the domains (data not shown). After the backward selection, all four domains contained two or more significant variables (P ≤ 0.157), which were combined in a multivariable model (Table 3). We analysed the five remaining significant variables (*P* ≤ 0.05) in a final multivariable model. In the final model, the demographic domain showed that older participants were less likely to RTW (OR 0.37, 95% CI 0.16–0.81). For the socio-economic and work-related domain, we found that participants who previously worked in a managerial position were more likely to return to work (OR 1.64, 95% CI 1.06–2.53). If participants received a partial or complete work disability benefit, they returned to work less often (OR 0.69, 95% CI 0.43–1.12 and OR 0.12, 95% CI 0.07–0.20). Within the domain of self-perceived ability, those who reported a good self-perceived work ability (OR 1.11, 95% CI 1.00–1.23) and a high possibility to RTW (OR 1.08, 95% CI 1.05–1.11) more often returned to work. The Hosmer and Lemeshow test was not statistically significant (*P*-value 0.82) in the final model, indicating that there was a good fit of the model. The Nagelkerke’s R2 was 0.37.

### Missing data

Missing data analyses showed that participants with an unknown RTW outcome differed significantly from the participants with a known RTW outcome. Participants with an unknown RTW outcome reported less good health, more complaints, less socio-economic status and less support (Additional file 1). Although the sensitivity analyses did not show any differences on regression coefficients in the multivariable and final model (Additional file 2), this meant that we could not completely rule out that the missing data was not merely a coincidence [32]. Therefore, we included only the results of the complete case analyses in this study; however, the results of the missing data analyses are presented in the supplementary materials for comparison (Additional files 1 and 2).

## Discussion

The main purpose of this study was to evaluate prognostic factors for RTW (return to work) after long-term sickness absence for workers with SHC (subjective health complaints). In our Dutch population, we found that receiving a work disability benefit after two years of sickness absence significantly predicted less RTW, and that high self-perceived possibilities for RTW resulted in more RTW after those two years for workers with SHC. These prognostic factors for RTW, as well as the number of workers that returned to work, were comparable for the reference group with other disorders; however, we found three additional factors that predicted RTW for the reference group: a lower age, a previous managerial position and a high self-perceived work ability. Our results suggest that non-health-related factors are more important than health-related factors in predicting RTW after long-term sickness absence.

Our results reveal that receiving a work disability benefit after two years of sickness absence is negatively related to the chances of returning to work successfully for workers with SHC. While some previous studies have supported that claim-related factors and compensation status are indeed associated with poorer health, longer sickness absence and less RTW [6, 33, 34] the literature in general has not paid much attention to this topic [35]. It is therefore difficult to determine whether it is poorer health status that leads to compensation and less RTW, or whether receiving compensation is a factor in RTW in and of itself. The literature that is available on this topic seems divided [36–39].

Our results seem to show an anti-therapeutic effect of disability compensation, as not the severity of the complaints but receiving work disability benefits had a negative influence on RTW for workers with SHC. The exact underlying mechanisms of this anti-therapeutic effect, however, are still difficult to determine [34]. Cassidy et al. [36] have argued that it could in part be explained by the theory of financial incentives, or secondary gain, as they found that removing the compensation increased health in workers with SHC. The explanation behind this hypothesis is that workers with SHC focus more on proving that their health complaints are real in the claim process at the expense of their RTW options because they are reluctant to RTW (i.e. less RTW willingness) for fear of losing their compensation and the validation of their being disabled [1, 37].

In contrast to the anti-therapeutic effect suggested by Cassidy et al. and others [36, 37], it is important to take into account that workers, regardless of their own feelings of recovery status, may be forced to RTW or to seek for another compensation because of financial necessity if they are not eligible for a work disability benefit. Although information about the course of those workers is scarce, the limited evidence on this topic revealed a high mental impact [38]. In addition, some studies have suggested that the process of applying for compensation can in fact make people more ill [39–41]. This is explained in terms of the distress caused by these claim settlement processes outweighing the possible positive effect of the expectation of gain [39, 40]. Importantly, this is irrespective of the underlying cause of the injury or the underlying pathology of the disease [41]. This is in line with the results of this study where the effect of a work disability benefit was not only valid for workers with SHC: we found comparable results for workers with other disorders. The results of this study corroborates the view that the process of applying for or receiving a disability compensation in and of itself may be a greater risk factor for permanent disability and less RTW than the severity and underlying pathology of the complaints and the health status in and of itself.

We also found that workers’ self-perceived possibilities for RTW was one of the most important factors for workers with SHC as well as for workers without SHC after long-term sickness absence. Young et al. [42] state that researchers have assumed that health-related factors, which were found as important factors for RTW in short-term sickness absence, remain the most relevant predictors for RTW after long-term sickness absence. However, a body of evidence supports the theory that for several chronic disorders, including persistent SHC, the importance of precipitating factors for RTW shifts during the sickness absence process [9, 10, 12, 43]. In fact, some studies on RTW after long-term sickness absence have indeed highlighted the workers’ own expectations for RTW as an important factor [9–12] and have shown that health-related factors become less important during sickness absence [13]. The present study indicates that this effect on RTW is indeed true for all workers: health-related factors, such as the underlying pathology and the severity of the disorder, became less relevant, and the non-health-related factors, such as the self-perceived expectations, became more relevant, for RTW after long-term sickness absence. In addition, factors that seem to be especially important for RTW for workers with other disorders than SHC, can also be classified as non-health-related factors. Contrasting to our expectations based on the literature beforehand [5–8], we found similar rates in RTW for workers with SHC and those with other disorders, which also corroborates the comparable results between these two groups in the present study.

### Strengths and limitations

The main strengths of this study were the use of broad data from participants from all regions of the Netherlands, which increases generalisability, and its prospective design. We asked workers to participate in the study when they were already sick-listed for two years, but just before their medical work disability assessment. We followed them for another two years, even if they were not granted a work disability benefit. This provided a unique opportunity to follow workers on RTW after long-term sickness absence, and to include the effect of a work disability benefit.

There are also some weaknesses in the present study. The first is the small response rate, due to the manner in which we included the participants. Because of stringent privacy regulations, we were not able to make a selection of workers beforehand. Therefore, we asked all 44,379 workers in the electronic database of UWV (The Dutch Social Security Institute) who were registered as sick listed for ≥84 weeks to participate in the study. They were asked to fill in a checklist without assistance and to respond only if they did not meet one of the criteria on the checklist and wanted to participate in the study. Out of the approached workers 9% responded, which is lower than average [44].

A second weakness follows from the first: we could not obtain more information about the non-responders as their data was not available. While it is certain that many workers who received a participation letter would normally not have been contacted, we may still assume – based on the high rate of non-response – that the characteristics of the study population may have caused some selection bias. It is possible that the non-responders were unhealthier than the responders, with possibly as result more positive outcomes in the present study. We did find, however, that the study sample was quite comparable with earlier studies on RTW for workers with other chronic diseases [13].

A further conceivable weakness is that as an outcome measure in research, data on sickness absence gathered from data files is preferable to data based on questionnaires [45]. However, questionnaires may still be considered a valuable source of information on overall sickness absence, and we had to use the questionnaires for the outcome measure due to the fact that this data on RTW after long-term sickness absence was not available in the UWV records.

In addition, missing follow-up questionnaires and missing answers in submitted questionnaires led to the exclusion of one-third of the respondents. However, the sensitivity analyses between the complete case analyses and the multiple imputation analyses for all participants showed comparable results on the regression coefficients in the final models. We take this to mean that there is missing at random (MAR), and that the data in the complete case analyses is robust, unselective and also representative for other workers.

### Implications for practice and future research

Based on the present study, support of RTW after long-term sickness absence has to be based especially on modifiable non-health-related factors, irrespective of the underlying pathology of the disorder. Previous studies have reported that delayed recovery could be improved by the implementation of more assistance, less medical assessments that have no therapeutic value, more personalized assessments, and more clarity in decision making in order to reduce the stressfulness for workers in the claim management process [39, 46, 47]. In addition, previous studies have reported that behaviour change interventions and interventions on self-efficacy may have the potential of optimizing the RTW process [48, 49]. However, more research is required to better examine the important underlying factors for positive RTW expectations and which interventions can help to change negative expectations for RTW into positive ones.

## Conclusion

Not receiving a work disability benefit and having positive expectations for RTW are the most important factors in RTW successfully after long-term sickness absence, both for workers with SHC as for those with other disorders. This suggests that non health-related factors are more important than health-related factors to predict RTW after long-term sickness absence.

## Supplementary information

**Additional file 1.** Missing data analyses of the baseline characteristics of the present study population.

**Additional file 2.** Multivariable logistic regression multiple imputation analysis (pooled data) of all potential predictors for participants with SHC^a^ and other disorders separately.

## Data Availability

The datasets generated and/or analysed during the current study are not publicly available due to possible individual privacy compromising but are available from the corresponding author on reasonable request.

## Abbreviations

CI: 95% confidence intervals
HADS: Hospital anxiety and depression scale
MUPS: Medically Unexplained Physical Symptoms
OR: Odds ratio
ORQ: Obstacles to return to work questionnaire
PHQ-15: Patient health questionnaire-15
PPS: Persistent Physical Symptoms
RTW: Return to work
SF-36: Short form health survey-36
SHC: Subjective health complaints
UWV: Dutch social security institute
VIF: Variance inflation factor
WAI: Work ability index
WAS: Work ability score
WBI: Work and well-being inventory
WI: Whitely index
