# Supplementary material for: Predicting return to work after long-term sickness absence with subjective health complaints: a prospective cohort study
Source: BMC Public Health. 2020 Jul 11;20:1095. doi: 10.1186/s12889-020-09203-5 (PMC7354686; doi:10.1186/s12889-020-09203-5)
Supplement: Supplementary file 1 — Additional file 1. Missing data analyses of the baseline characteristics of the present study population. [file 12889_2020_9203_MOESM1_ESM.docx]

***Missing data analyses of the baseline characteristics of the present study population***

|  |  | **Cases with missing outcome^a^ (N=655)** | | **Cases with no missing outcome^b^ (N=1276)** | | **Chi-square or T-test** |
| --- | --- | --- | --- | --- | --- | --- |
| **Domains** | **Categories/ Ranges** | **Mean/N^c^** | **SD^d^/%** | **Mean/N** | **SD/%** | **P-value** |
| ***Demographic*** | | | | | |  |
| Age in years | 18-34 | 84 | 13% | 70 | 6% | 0.00 |
|  | 35-44 | 97 | 15% | 166 | 13% |  |
|  | 45-54 | 235 | 36% | 435 | 34% |  |
|  | 55-65 | 239 | 36% | 605 | 47% |  |
| Gender | Male | 302 | 46% | 652 | 51% | 0.04 |
| Marital status | Married or partner | 433 | 66% | 949 | 74% | 0.00 |
| Breadwinner of the family | Yes | 398 | 61% | 817 | 64% | 0.91 |
| Land of birth | The Netherlands | 529 | 81% | 1163 | 91% | 0.00 |
| Educational level | None/Primary school | 102 | 16% | 112 | 9% | 0.00 |
|  | Secondary school | 251 | 38% | 507 | 40% | - |
|  | High school | 219 | 34% | 413 | 32% | - |
|  | Bachelor/Master | 81 | 12% | 242 | 19% | - |
| ***Socio-economic and work-related*** | | | | | |  |
| Collar job | Blue | 220 | 36% | 405 | 33% | 0.33 |
|  | White | 196 | 32% | 431 | 35% | - |
|  | Pink | 197 | 32% | 387 | 32% | - |
| Employer | Yes | 202 | 33% | 463 | 38% | 0.04 |
| Usual working time in hours | 4-60 | 32.49 | 10.68 | 32.50 | 10.77 | 0.98 |
| Work schedule | Regular | 399 | 61% | 825 | 65% | 0.12 |
| Managerial position | Yes | 135 | 21% | 273 | 21% | 0.68 |
| Job demands | Psychological | 119 | 18% | 301 | 24% | 0.01 |
|  | Physical | 243 | 37% | 414 | 33% | - |
|  | Psychological and physical | 290 | 45% | 554 | 44% | - |
| Stressors | 16-64 | 39.15 | 9.53 | 38.23 | 9.31 | 0.05 |
| Support | 21-84 | 57.28 | 12.70 | 58.85 | 12.76 | 0.01 |
| Previous absenteeism same reason | Yes | 331 | 52% | 601 | 48% | 0.09 |
| Work disability benefit | No | 152 | 25% | 197 | 16% | 0.00 |
|  | Partial | 105 | 17% | 310 | 26% | - |
|  | Complete | 355 | 58% | 703 | 58% | - |
| ***Health-related*** | | | | | |  |
| Use of specialist care last 2 years | Yes | 544 | 83% | 1093 | 86% | 0.13 |
| Use of psychiatric care last 2 years | Yes | 316 | 48% | 620 | 49% | 0.89 |
| Use of medication | Yes | 575 | 88% | 1143 | 90% | 0.25 |
| Depressive disorder | No | 190 | 29% | 465 | 37% | 0.00 |
|  | Maybe | 147 | 23% | 288 | 23% | - |
|  | Yes | 315 | 48% | 520 | 41% | - |
| Anxiety disorder | No | 218 | 33% | 509 | 40% | 0.00 |
|  | Maybe | 132 | 20% | 291 | 23% | - |
|  | Yes | 304 | 47% | 475 | 37% | - |
| Severity of complaints | Mild | 165 | 25% | 390 | 31% | 0.01 |
|  | Moderate | 212 | 32% | 430 | 34% | - |
|  | Severe | 278 | 42% | 456 | 36% | - |
| Physical Health | 0-100 | 30.36 | 8.96 | 31.26 | 9.62 | 0.04 |
| Mental Health | 0-100 | 33.20 | 13.33 | 34.56 | 13.61 | 0.04 |
| Health change comparing last year | Worse | 359 | 55% | 695 | 55% | 0.11 |
|  | Same | 203 | 31% | 361 | 28% | - |
|  | Better | 89 | 14% | 218 | 17% | - |
| Hypochondria | Yes | 489 | 75% | 872 | 69% | 0.00 |
| Symptom scale | 20-80 | 47.87 | 12.65 | 45.16 | 11.91 | 0.00 |
| Coping strategies | 17-68 | 42.60 | 9.70 | 42.77 | 9.56 | 0.72 |
| SOLK | Yes | 127 | 20% | 213 | 17% | 0.17 |
| ***Self-perceived ability*** | | | | | |  |
| Return to work expectation | Yes or maybe | 462 | 71% | 858 | 67% | 0.11 |
| Disability | 7-28 | 24.78 | 3.89 | 24.89 | 3.65 | 0.56 |
| Work ability in general | 0-10 | 2.27 | 2.13 | 2.18 | 1.93 | 0.41 |
| Work ability in context of work load | 0-10 | 4.16 | 1.62 | 4.26 | 1.61 | 0.20 |
| Possibilities for returning to work | 0-36 | 9.39 | 7.89 | 9.67 | 8.14 | 0.50 |

Footnotes:

^a^No fully documented work status during follow-up

^b^Fully documented work status during follow-up

^c^N = Number

^d^SD = Standard Deviation
