# Supplementary material for: Predicting return to work after long-term sickness absence with subjective health complaints: a prospective cohort study
Source: BMC Public Health. 2020 Jul 11;20:1095. doi: 10.1186/s12889-020-09203-5 (PMC7354686; doi:10.1186/s12889-020-09203-5)
Supplement: Supplementary file 2 — Additional file 2. Multivariable logistic regression multiple imputation analysis (pooled data) of all potential predictors for participants with SHCa and other disorders separately. [file 12889_2020_9203_MOESM2_ESM.docx]

## ***Multivariable logistic regression multiple imputation analysis (pooled data) of all potential predictors for participants with SHC^a^ and other disorders separately***

|  |  | **SHC^a^** | | **Other disorders** | | |
| --- | --- | --- | --- | --- | --- | --- |
| **Domains** | **Categories/Ranges** | **OR** | **95% CI** | **OR** | **95% CI** | |
| ***Demographic*** | | | | | | |
| Age in years | 18-34 |  |  | **Reference** | | |
|  | 35-44 |  |  | **0.75^b^** | **0.34-1.67** | |
|  | 45-54 |  |  | **0.61** | **0.28-1.33** | |
|  | 55-65 |  |  | **0.32** | **0.15-0.68** | |
| Land of birth | The Netherlands | Reference | | Reference | | |
|  | Another country | 2.18 | 0.76-6.24 | 1.33 | 0.76-2.32 | |
| Educational level | None/Primary school | Reference | | Reference | | |
|  | Secondary school | 1.35 | 0.32-5.79 | 1.09 | 0.53-2.24 | |
|  | High school | 2.32 | 0.55-9.78 | 1.26 | 0.61-2.58 | |
|  | Bachelor/Master | 2.60 | 0.59-11.55 | 1.01 | 0.47-2.18 | |
| ***Socio-economic and work-related*** | | | | | | |
| Employer | No |  | | Reference | | |
|  | Yes |  | | 1.21 | | 0.84-1.74 |
| Manegerial position | No |  | | **Reference** | | |
|  | Yes |  |  | **1.65** | **1.10-2.47** | |
| Work disability benefit | No | **Reference** | | **Reference** | | |
|  | Partial | **0.65** | **0.27-1.58** | **0.73** | **0.47-1.13** | |
|  | Complete | **0.22** | **0.09-0.57** | **0.13** | **0.08-0.22** | |
| ***Health-related*** | | | | | | |
| Use of medication | No |  | | Reference | | |
|  | Yes |  |  | 1.01 | 0.61-1.68 | |
| Physical Health | 0-100 |  |  | 1.01 | 0.99-1.03 | |
| Health Change comparing last year | Worse | Reference | | Reference | | |
|  | Same | 0.45 | 0.17-1.21 | 0.97 | 0.63-1.48 | |
|  | Better | 1.17 | 0.46-2.94 | 0.78 | 0.46-1.33 | |
| ***Self-perceived ability*** | | | | | | |
| Work ability in general | 0-10 |  |  | **1.12** | **1.01-1.25** | |
| Possibilities for returning to work | 0-36 | **1.06** | **1.01-1.11** | **1.08** | **1.05-1.10** | |

Footnotes:

^a^SHC = Subjective Health Complaints

^b^OR = Odds ratio

^c^95% CI = 95% confidence intervals

^d^Numbers in bold had a p-value of ≤0.05 and were combined the final model
